# Antimalarial Efficacy of Hydromethanolic Root Extract and Solvent Fractions of *Urtica simensis* Hochst. ex. A. Rich. (Urticaceae): An Experimental Study on *Plasmodium berghei*-Infected Mice

**DOI:** 10.1155/2022/6702733

**Published:** 2022-03-29

**Authors:** Woretaw Sisay, Yared Andargie, Mulugeta Molla

**Affiliations:** Department of Pharmacy, College of Health Sciences, Debre Tabor University, Debre Tabor, Ethiopia

## Abstract

**Background:**

Despite modern therapeutic armamentariums, malaria remains a 21st century public health menace. The issue of combating malaria is the ever-growing resistance to high-tech medications in which novel phytomedicines are highly demanding, a rapidly expanding research avenue. In Ethiopian folklore medicine, *Urtica simensis* has been used to treat malaria by drinking its juice after the dry roots have been mashed and combined with water. Hitherto, no in vivo study has been reported in the literature so far. To substantiate this folkloric claim, the present work herein was done.

**Methods:**

An acute oral toxicity study was conducted as per the standard protocol. To rule out, the extract's inherent potential effects on bodyweight, basal body T^o^, and PCV changes were tracked for two weeks. A four-day suppressive model and a curative assay model were utilized to investigate the antimalarial activity of the plant. Percent parasitemia suppression, packed cell volume, mean survival date, bodyweight, and rectal body temperature were used to determine antimalarial activity.

**Result:**

An acute toxicity study reveals that *Urtica simensis* was atoxic at a dose of 2000 mg/kg. It also affirms that *U. simensis* is free from intrinsic potential effects of interfering with bodyweight, temperature, and packed cell volume evolution. Both crude extract and its solvent fractions at all test doses exerted significant (*P* < 0.001) inhibition of parasitemia as compared to the control group. CF400 mg/kg provided the greatest chemosuppressive effect (79.24%). In a curative experiment, crude extract and CF were able to prevent the cardinal indications of *P. berghei*-induced malaria, such as weight loss, hypothermia, parasitemia, and anemia. Both crude extracts and their solvent fractions prolong survival dates.

**Conclusion:**

The antimalarial activity of the crude extract and its solvent fractions was promising, confirming previous assertions. As a result, more research studies into chemical entities may be required.

## 1. Introduction

### 1.1. Background

Although malaria is a preventable and curable malady, it still remains a major public health menace. In 2019, there were an estimated 229 million malaria cases worldwide, with 409000 fatalities. In 2019, Africa accounted for more than 94% (215 million) of global cases and deaths (386,000). Due to a weakened immune system, children under the age of five and pregnant women are the most vulnerable [1]. Malaria infected 33.2 million pregnant women in Africa in 2019, accounting for 35% (11.6 million) of all infections in 2019. Children under the age of five will account for 67% of all malaria-related deaths worldwide in 2019 [2].

It is one of the top 10 causes of mortality in Ethiopia, accounting for 2% of all deaths [3]. The parasites *Plasmodium falciparum* and *Plasmodium vivax* are by far the most common and widely dispersed. *P. vivax* was responsible for only 2.3% of those cases [4]. Despite the fact that both malaria cases and deaths have decreased in recent years, progress is endangered by the emergence of insecticide-resistant mosquitoes, drug-resistant malaria parasites, and a lack of a successful and effective licensed malaria vaccine [5].

Since the dawn of time, mankind has employed medicinal plants to combat ailments such as malaria. According to the World Health Organization, about three-quarters of the world's population receives healthcare from plants and their extracts. Traditional medicine is used to meet some of the most basic healthcare needs in a number of African, Asian, and Latin American countries [6]. Over 80% of the population in Ethiopia relies on traditional medicine to cure a variety of illnesses because of the cultural acceptability of healers and the relative affordability of traditional medicine [6, 7]. Furthermore, botanicals account for more than a quarter of all modern pharmaceutical medications [8].

Many herbal plants around the world have antimalarial properties due to secondary metabolites such as tannins, alkaloids, saponins, flavonoids, steroids, and terpenoids [9]. Both quinine and artemisinin derivatives, now used to treat severe falciparum malaria, are natural products. In vitro and in vivo studies looking for novel antimalarial medicines of natural origin were inspired by the development of these two key treatments from natural sources [10].

Traditional herbalists use the plant without assessing and optimizing the amounts of physiologically active components. At a dose of 200 mg/kg, extract fractions from *Withania somnifera* had a considerable suppressive effect on malaria [11]. Similarly, crude extracts of *C. myricoides*, *D. angustifolia*, and *A. debrana* have potent anti-*P. berghei* properties in mice. These are only a few of the many therapeutic plants whose pharmacological effects have been approved [12].

*Urtica simensis* belongs to the family Urticaceae and genus *Urtica*. The Urticaceae family, also known as the nettle family, is made up of 48 genera and about 2000 species of plants. The tropical and subtropical regions are home to the majority of these plant species. *Urtica* is derived from the Latin word “urere,” which means “to burn.”

After having been stung by nettles, humans are generally able to recognize them [13, 14]. Medicinally, several *Urtica* species (particularly *Urtica dioica* L.) are used to cure a number of maladies. *Urtica simensis* is an indigenous nettle species found only in Ethiopia. The plant grows throughout the year at 1500–3500 m above sea level in Ethiopia's highlands, particularly in the North and South Gondar, North and South Wello, North Shewa, Wag Hamra, highlands of Sidama zone in the Southern region, and Arsi zone of Oromia region. It is mostly seen in the vicinity of dwellings. The plant is commonly known as “Nattle,” “sama” (Amharic) [15]. *Urtica simensis* is a dark green perennial wild plant that has been used for food in the past, especially during droughts. It has a lot of promise and can help with food security by meeting human nutritional needs [16, 17]. *Urtica simensis* is well known for its stinging hairs that grow beneath the stems and on the lower leaf surface. The plant is completely covered in stinging hairs. Herbal nettles are one meter tall, dioecious, erect, and nonbranched. The leaves are opposite and simple, with united stipules and interpetiolar lengths of 0.5–1 cm. The leaves are slightly cordate at the base, broad and sharp at the apex, and serrated at the border. *Urtica simensis* has unisexual, regular blooms, and the fruit is about 2 mm long [18].

Different portions of the *U. simensis* plant are used for the treatment of various illnesses and ailments, according to ethnobotanical studies on traditional medicinal usage of the plant in Ethiopia. To treat gonorrhea, the root and leaf parts of the *U. simensis* plant are powdered, mixed with water, and the filtrate is consumed. Washing the diseased area of the body once a day with a root and leaves infusion is also used to cure gonorrhea [15, 19]. The plant's leaves and young twigs, on the other hand, are crushed, and the resulting leaf juice is creamed with butter and applied topically to wound infections. In two varieties of preparation, the fresh leaves of *U. simensis* are used to treat gastritis. As a result, the leaves are roasted, ground into smaller bits, and the juice is consumed orally, or the fresh leaves can be prepared and eaten with “injera” [15, 20]. It was said to be used to treat acute stomach pain (by drinking the sap orally) and body edema (heat and put the leaf on the affected area topically) 16, a common cold (fresh root ground and taken orally) [21], Rh-factor, and heart failure (number 22) (fresh leaf steam vapor allowed to enter nasally and fumigated whole body) [22]. Furthermore, after being crushed and dried, the plant's root is mixed with fresh water and consumed along with a large amount of milk to treat malaria [23]. Its leaf extract possesses antibacterial, antifungal [24], antidiabetic [25], and cardioprotective [26] properties. The antimalarial activity of this plant's roots has not been scientifically confirmed. The purpose of this study is to test the antimalarial activity of both the crude extract and solvent fractions of *Urtica simensis* root. The study also aimed to shed light on the phytochemicals responsible for the plant's activity by examining the plant's phytochemical composition. The outcomes of this study may help to develop new antimalarial drugs that solve existing antimalarial drug concerns.

## 2. Materials and Methods

### 2.1. Materials

#### 2.1.1. Drugs and Chemicals

The following drugs, chemicals, and instruments were used in the experiment during the study period: absolute methanol (ReAgentchem. Ltd., India), 0.9% normal saline (Cadila Pharmaceuticals, Bengaluru, India), distilled water (EPharm, Ethiopia), chloroform, ethyl acetate, n-butanol, examination glove, 0.5% trisodium citrate, 95% sulfuric acid (Fisher Scientific, UK), Mayer's reagent, Dragendorff's reagent, glacial acetic acid (Lobe Chemi, India), benzene (Nice Laboratory Reagent, Kerala, India), ammonia solution, FeCl_3_ (Super Tek Chemicals), 10% ammonium hydroxide (Rankem, Mumbai, India), 0.01 N sodium hydroxide (Central Drug House India), acetic anhydride and Mayer's reagent (May and Baker Ltd., England), dimethyl sulfoxide (DMSO), chloroquine 500 mg tablet, Giemsa stain solution, and syringe which is analytical graded (Addis Ababa Pharmaceutical and Chemical Suppliers).

#### 2.1.2. Instruments, Apparatus, and Supplies

The mini-orbital shaker (MaxQ 2000, USA), rotary evaporator (rotary evaporator RE300, UK), digital weighing balance (Mettler Toledo, Switzerland), separatory funnel, lyophilizer (Wagtech Jouan Nordic DK-3450), deep freezer, light microscope (Olympus N-120A, Philippines), microscope slide (LANCONCO, Freeze Dry System, USA), grinder (KZ-III, Wuhan, China), Whatman filter paper (number 1) (Schleicher and Schuell Microscience GmbH, Germany), permanent marker, microhematocrit centrifuge (Hettich, Germany), microhematocrit reader, and digital thermometer were used in the experiment during the study period.

#### 2.1.3. Experimental Animals

Healthy Swiss albino mice (aged 8–12 weeks, weighting 27–35 g, either sex) were used. They were housed in raised mesh bottom cages to prevent coprophagy in conventional laboratory settings (25.2°C, 12 h light and dark cycle). They were fed conventional pelleted feed and free access to water. They were acclimatized for 5 days before being divided into groups at random in each phase of the experiment. All procedures in this study were carried out in accordance with local and international ethical guidelines, including the Basel Declaration [27], ICLAS Ethical Guideline [28], and European Union directive [29].

### 2.2. Methods

#### 2.2.1. Collection and Authentication of Plant Materials

The root of *Urtica simensis* was collected at Debre Tabor district, Amhara Regional state, about 655 km northwest of Addis Ababa in January 2020. Botanical identification and authentication were done by an expert taxonomist, Dr. Getinet Masresha, at Department of Biology College of Natural and Computational Sciences, University of Gondar, and a voucher specimen Wor3/2020 was kept there for future reference.

#### 2.2.2. Preparation and Extraction of Plant Materials

The roots were first washed under flowing tap water to eliminate dirt or dust and then dried in the shade in the pharmacology laboratory. Using a grinder, it was mechanically chopped into coarse powder. Until extraction, the powder sample was weighed and stored in airtight containers.

#### 2.2.3. Extraction Procedure for Crude Extract

This research made use of a 80% methanol because it can extract a wide variety of polar and moderately polar compounds [30]. The 80% methanol crude extract of root of *U. simensis* was prepared by the cold maceration technique. After weighing 1 kg of coarsely powdered root with a sensitive digital weighing balance, it was steeped in an 80% methanol Erlenmeyer conical flask. After that, the flask was shaken at 120 rpm for 72 h at room temperature with a mini-orbital shaker. A Whatman filter paper (No. 1) was used to refine the filtrate after it had been passed through two muslin cloth filter stages. A fresh solvent of 80% methanol was used to remacerate the leftover residue or marc twice for a total of six days to maximize production. After successive filtration, methanol was removed by a rotary evaporator set to 40°C and lyophilized to remove water. The % yield was computed by the following equation and kept at −20°C until used [31].

(1) $$\begin{array}{c} \text{\% yield}=\;\frac{\text{Weight of crude hydromethanolic extract obtained}}{\text{Weight of sample powder used for extraction}}*1000 \end{array}$$

#### 2.2.4. Fractionation of the Crude Extract

In a separatory funnel system, methanol extracts of the root of *U. simensis* underwent fractionation using a solvent-solvent partitioning technique using solvents of different polarities. Because, this process is critical as the initial step in separating chemicals from crude natural product extracts on a large scale [30]. It was done by initially measuring 85 g of crude extract, transferring it in to a clean Erlenmeyer conical flask containing 300 ml of distilled water, allowed to suspend, and then shaken to dissolve in solvent. After transferring the mixture to a separating funnel, 300 ml of chloroform was added. The chloroform fraction was collected in a separate flask. Furthermore, 300 ml of chloroform was added twice as portrayed above and finally concentrated and dried using a rotary evaporator and an oven, respectively. Ethyl acetate (300 ml) was poured into the marc thrice consecutively upon collecting the filtrate. Then, separated ethyl acetate filtrate was concentrated and dried using a rotary evaporator and an oven, respectively. Upon lyophilization, the leftover aqueous fraction was retrieved. Finally, the % yield was computed by the following equation:

(2) $$\begin{array}{c} \text{\% yield}=\;\frac{\text{Weight of solvent fractions obtained}}{\text{Weight of crude extracts used}}*100 \end{array}$$

#### 2.2.5. Determination of Acute Oral Toxicity Potential

The median lethal dose of root extract of *U. simensis* was predicted in female mice, following the OECD guideline 425 with some modifications [32]. In brief, twelve animals of 8–12 weeks were used in experimentation. Then, they were arbitrarily divided into two groups. Following a 4 h fast, all animals were weighed, and doses were established depending on their physical weight. To do this, a limit dose of 2 g/kg extract dissolved in 2% tween 80% (TW80) was administered PO to a single female mouse and observed for gross toxicity and deaths within 24 h. Then, five other mice were sequentially treated and observed continuously for 2 weeks for toxicity patterns [33]. The other six animals of the control group were treated with TW80 10 ml/kg with identical scheme of administration. Likewise, bodyweight, rectal body temperature, and packed cell volume changes were recorded before treatment (baseline) and daily for two weeks.

### 2.3. In Vivo Antimalarial Probing

#### 2.3.1. Parasite Inoculation

Ethiopian Health and Nutrition Research Institute (EHNRI) was the source of the chloroquine-sensitive *Plasmodium berghei* (ANKA) strain. This parasite enables us to produce a rodent model of malaria that mimics human malaria infection [34, 35]. The parasites were kept alive by serially transferring blood every week. Donors were albino mice which attains a 20–30% parasitemia level. Upon decapitation, their blood was drawn into a test tube. After dilution of blood with normal saline (0.9%), each mouse received 0.2 ml of diluted blood with 1 × 10 [7] *Plasmodium berghei*-infected red blood cells [36].

#### 2.3.2. Grouping of Animals and Experimental Procedure

Female animals weighing 27–35 g were arbitrarily divided into five groups of 6 per group. Groups I–III received 100 mg/kg (US100), 200 mg/kg (US200), and 400 mg/kg (US400), respectively. Group IV and V received distilled water or TW80 (1 ml/100 g) and chloroquine phosphate 25 mg/kg (CQ25), respectively.

In a study of fractions, animals were randomly assigned into three treatment groups and two controls, six animals per group. Negative controls received the vehicle used for reconstitution. Treatment groups received 100 mg/kg, 200 mg/kg, and 400 mg/kg of corresponding fractions. Positive controls were treated with chloroquine. Models employed for this study were tested by Peter's test (4-day suppression test) and Rane's (curative) test [37].

#### 2.3.3. Peter's Test

Peter' test was used to determine the treatment's schizonticidal activity using the approach provided by Peter et al. [38]. Three hours after infection, on day 0, treatment was initiated and then continued daily until day 3. Each animal had a sample taken from its tail after therapy was completed in order to measure parasitemia and the percentage of inhibition.

#### 2.3.4. Curative Assay Model

The methodology described by Ryley and Peters was used to investigate the curative ability of the methanolic crude extract and the most active solvent fraction of *U. simensis* in Peter's test [39]. Mice were randomly divided into four groups 72 hours after infection and given the appropriate doses once daily for 4 days. The parasitemia level was assessed daily for five days employing a thin blood film dyed with Giemsa.

#### 2.3.5. Mean Survival Time Determination

Over the course of 30 days (D0–D29), the average survival time (days) of mice was calculated arithmetically, as [40]

(3) $$\begin{array}{c} \text{MST}=\frac{\text{Sum of survival time of all mice in a group }\left(\text{days}\right)}{\text{sum of mice in the group}} \end{array}$$

#### 2.3.6. Packed Cell Volume Determination

To anticipate the effectiveness of the test extracts in halting hemolysis caused by rising parasitemic levels, the number of packed cells was analyzed. Each mouse's tail was scraped, and blood drawn into heparinized capillary tubes. After filling the capillary tubes with blood up to three-fourths of their volume, they were then sealed shut with sealing clay. Afterward, the sealed end of the tubes was placed in a microhematocrit centrifuge with 11000 rpm and spun for five minutes. Using a basic microhematocrit reader, we were able to determine PCV levels. The ratio of RBCs to plasma is assessed before and after parasite inoculation and therapy using the following formula:

(4) $$\begin{array}{c} \text{PCV }=\frac{\text{Erythrocytes count in a given volume of blood}}{\text{Total blood volume }} \end{array}$$

#### 2.3.7. Appraisal of Parasitemia Level

On day 4 for Peter's test and on days 3–7 for Rane's test, thin smears of blood were retrieved from the tail of individual animal, fixed for 15 min in pure methanol, and dyed for 15 min with 10% Geimsa stain at pH 7.2 for the smears on microscope slides (76 × 26 mm). Using distilled water, the stained slides were gently cleaned and allowed to air dry at room temperature. Each mouse had two stained slides analyzed using an Olympus microscope. The mean parasitemia was determined by considering the three separate fields on each slide, as shown in the graph [41].

(5) $$\begin{array}{c} \text{\% parasitemia}=\frac{\text{Number of afflicted red blood cells}}{\text{Whole red blood cells }}*100 \end{array}$$

Ultimately, the potential suppressive effect of the treatments was compared to that of infected but untreated groups, and parasitemia suppression was computed using the following equation.

(6) $$\begin{array}{c} \text{\% suppression}=\frac{\text{Average parasitemia of infected control}-\text{average parasitemia of the treated group}}{\text{Average parasitemia of infected control}}*100 \end{array}$$

#### 2.3.8. Evaluation of Bodyweight and Rectal Temperature Flux

Bodyweight of animals was evaluated preinfection (day 0) and after treatment (day 4) in case of Peter's test. Furthermore, rectal T^o^ was evaluated by a rectal thermometer preinfection at day 0 and on day 4 after treatment. Bodyweight and rectal T^o^ were evaluated in day 3 and day 7 by using Rane's assay.

(7) $$\begin{array}{c} \text{Mean bodyweight}=\frac{\text{Total weight of mice in a group}}{\text{Total number of mice in that group}}*100 \end{array}$$

### 2.4. Preliminary Phytochemical Profiling

Standard assays were employed to conduct qualitative phytochemical studies on *Urtica simensis* root extracts [42–44].

### 2.5. Data Analysis

The study's findings are summarized as mean ± SEM. One-way analysis of variance (ANOVA) was performed followed by the Tukey HSD post hoc test for multiple comparisons between tests. Two-way analysis of variance was performed followed by Bonferroni's post hoc test for tests in outcome variable before and after treatment using IBM SPSS for window (Version 24.0) statistical package. *P* < 0.05 was considered statistically significant at a 95% confidence interval.

### 2.6. Data Quality Assurance

Data quality was maintained by categorizing experimental laboratory animals by a random sampling statistical technique, collecting data of all indicators blindly, maintaining and applying standard procedures consistently, and using scientifically labeled instruments.

## 3. Results

This study authenticated the percentage yield, phytochemical ingredients, the acute oral toxicity, and the antimalarial activity of 80% hydromethanolic crude extract and solvent fractions of roots of *U. simensis* against *P. berghei* in mice.

### 3.1. Extraction Yield of Hydromethanolic Crude Extract and Solvent Fractions

Traditionally, the roots of *U*. *simensis* are taken after being crushed and mixed with fresh water for the treatment of malaria. Since the role of water is as a vehicle, 80% MeOH was used so as to extract most of the constituents in the roots that might explain its activity. Generally, hydroalcohols are related with higher extract yields [45]. Accordingly, 80% methanol root extract of *U. simensis* afforded a dark bluish powder with percentage yield of 153 g (15.3% w/w) and stored in a refrigerator at 4°C until use (Table 1).

### 3.2. Preliminary Phytochemical Profiling

The preliminary phytochemical screening of 80% hydromethanolic crude extract and solvent fractions of roots of *U. simensis* for the probable presence of different secondary metabolites are given in Table 2. These qualitative tests were done as per standard test protocols. These tests divulge the existence of terpenoids, tannins, saponins, flavonoids, alkaloids, saponins, and phenolic compounds.

### 3.3. Acute Oral Toxicity Study

The acute toxicity study showed that the root extract caused no mortality at 2000 mg/kg within the first 24 h and for the next 2 weeks study period. Physical and behavioral observations of the experimental mice also revealed no visible signs of acute toxicity such as lacrimation, loss of appetite, tremors, sweating, piloerection, enuresis, salivation, seizure, trembling, and diarrhea. In light of this, the mean lethal dose of the candidate plant extract (LD_50_) was beyond 2000 mg/kg. Eventhough it lacks significant acute toxicity in mice at the dose level used in this experiment, in further studies, subacute toxicity needs to be executed. Based on OECD-425 guideline, if the limit dose (2000 mg/kg) is deemed atoxic, 1/10th of 2000 mg/kg (200 mg/kg) medium dose, 1/20th of 2000 mg/kg (200/2 = 100 mg/kg) low dose, and 1/5th of 2000 mg/kg (200 × 2 = 400 mg/kg) high dose can be applied in the study.

#### 3.3.1. Effect of *U. simensis* on Bodyweight, Basal Body Temperature, and Packed Cell Volume of Normal Mice

**Figure 1:**
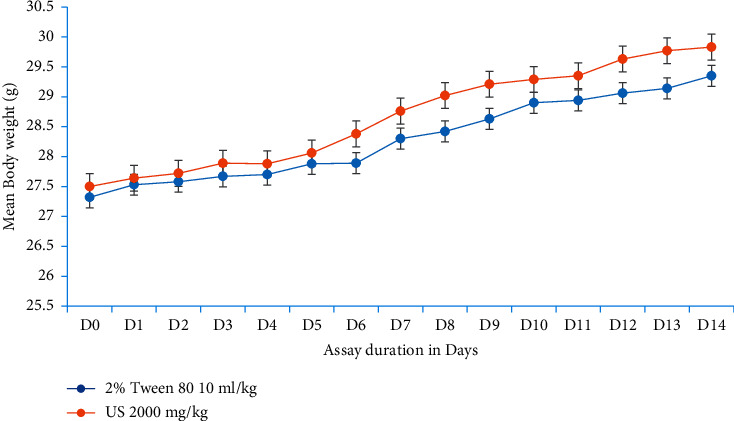
Evolution of mouse bodyweight over the course of an experiment. Data are expressed as mean ± SEM (*n* = 6). D, days; US, *Urtica simensis*.

**Figure 2:**
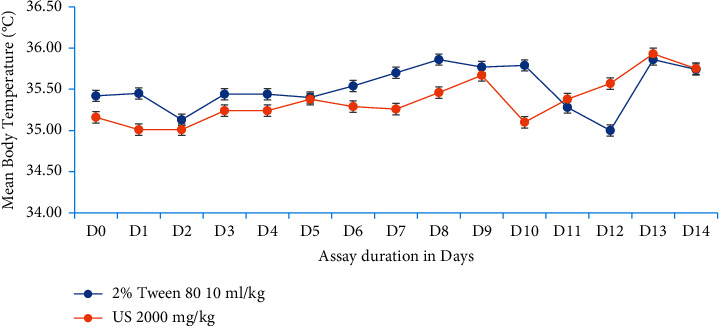
Progression of mouse basal body temperature with respect to experimentation duration. Data are expressed as mean ± SEM (*n* = 6). D, days; US, *Urtica simensis.*

**Figure 3:**
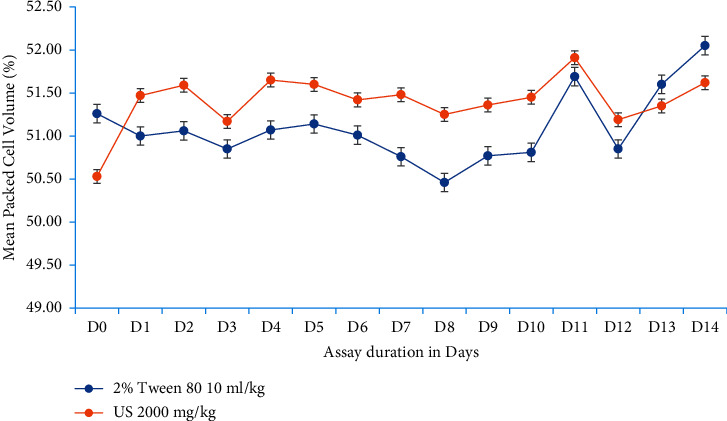
Progression of mouse packed cell volume with respect to experimentation duration. Data are expressed as mean ± SEM (*n* = 6). D, days; US, *Urtica simensis.*

In the next set of experiments, *U. simensis* at 2000 mg/kg did not bring neither bodyweight loss, basal body temperature reduction, nor hemolysis in test mice compared to control ones as well as to its baseline value. In brief, bodyweight in the test group has been increased further throughout the assay period in line with the control group, inferring that the extract does not affect bodyweight negatively by itself (Figure 1). While in case of basal body temperature and packed cell volume, there is no overt difference (*P* > 0.05) in between groups and before and after treatment periods in both test groups (Figures 2, 3).

### 3.4. In Vivo Antiplasmodial Efficacy Studies

#### 3.4.1. Chemosuppressive Effect in the 4-Day Suppressive Test

The effect of hydromethanolic crude extract and its solvent fractions of root of *U. simensis* at different dose levels on parasitemia and mean survival time of mice infected with chloroquine-sensitive *P. berghei* are given in Table 3. Briefly, the results were expressed as mean percentage parasitemia, mean percentage chemosuppression, and mean survival time in days in comparison with the respective negative control groups.

Blood smears from the untreated control on D4 (5th day after parasite inoculation) disclosed nil parasite reduction with a mean parasitemia of 41.03 ± 0.95, 39.25 ± 2.34, 42.36 ± 0.94, and 36.79 ± 2.01 parasites in blood assayed parallelly with crude extract, CF, EF, and AF, respectively. Crude extract produced a dose-dependent chemosuppressive effect when tested against early infection, with daily doses of 100, 200, and 400 mg/kg, causing 55.23%, 68.00%, and 77.48% chemosuppression 96 h postinfection, respectively. The standard drug, chloroquine, cleared the parasite (100% suppression) by day 4 when used at 25 mg/kg per identical condition. As given in Table 3, all three fractions significantly suppressed parasite count in the 4-day suppressive test. Of these, the aqueous fraction showed the lowest chemosuppression. All fractions at all doses exerted significant (*p* < 0.001) inhibition of parasitemia as compared to the control group. The highest chemosuppressive effect (79.24%) was offered by chloroform fraction, CF400. The reference agent, chloroquine, cleared parasitemia to undetectable state on the fifth day from a thin blood film with no observed mortality in the group after 30 days.

The mean survival rate of infected mice treated with the candidate plant crude extract significantly prolonged at all dose points (*P* < 0.001) of the extract except US100. However, this is deemed lower than the outstanding agent (chloroquine phosphate 25 mg/kg) that markedly (*P* < 0.0001) prolong survival of mice. Eventhough all solvent fractions were able to prolong life expectancy of mice at all dose points, the longest survival date (17.27 (±0.98) days) was recorded in CF400. On the contrary, AF100 failed to reach statistical significance in prolonging survival date. Moreover, group of mice treated with the standard agent was viable throughout the study period (30.00 ± 0.00 days), except positive control mice in AF (29.83 ± 0.11 days). Eventhough both crude extract and CF were able to prolong life of animals, it is markedly lower (*P* < 0.001) than the standard agent ensued.

#### 3.4.2. Effect of *U. simensis* Crude Extract and Solvent Fractions on Packed Cell Volume in the Suppressive Test

**Figure 4:**
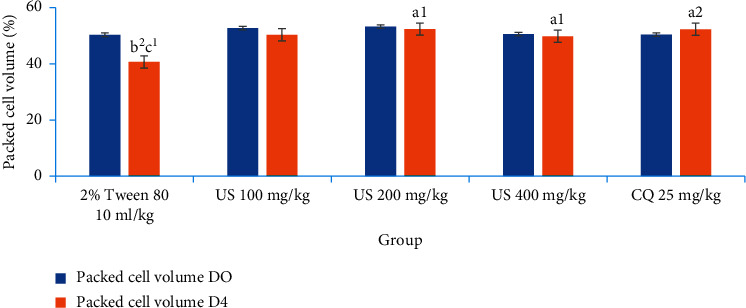
The effect of hydromethanolic crude root extract of *U. simensis* on packed cell volume of *P. berghei*-infected mice in the 4-day suppressive test. Data are expressed as mean ± SEM (*n* = 6). ^a^Compared to negative control. ^b^Compared to the standard. ^c^Compared to baseline. ^1^*P* < 0.05; ^2^*P* < 0.01. US, *Urtica simensis*.

As shown in Figure 4, in comparison to the control group, US200 and US400 significantly (*P* < 0.05) averted reduction in PCV in mice at all doses, but US100 fell short to reach the statistical significance which showed a significant reduction in PCV (−6.71 ± 6.60). Eventhough there was a decrease in PCV of crude extract treated animals, this reduction failed to reach the statistical significance between day 0 and day 4 compared to the vehicle-treated group. In the contrary, the vehicle-treated group of animals culminated a significant decrease in PCV on day 4 as compared to the baseline. The vehicle-treated group showed appreciable reduction in PCV 96 hours postinfection. In the contrary, the group of mice treated with the standard agent revealed an increase in volume packed cells (2.69 ± 7.83).

No apparent difference was observed among the three doses of the crude extract and solvent fractions in protecting the packed cell volume of the mice. The standard drug, in comparison with all doses of the crude extract, did not show notable differences in packed cell volume protection.

**Figure 5:**
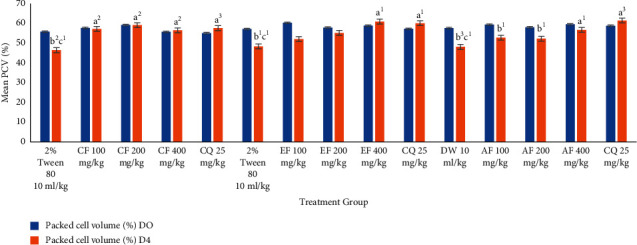
The effect of solvent fractions of root of *U. simensis* on packed cell volume of *P. berghei*-infected mice in the 4-day suppressive test. Data are expressed as mean ± SEM (*n* = 6). ^a^Compared to negative control. ^b^Compared to the standard. ^c^Compared to baseline. ^1^*P* < 0.05; ^2^*P* < 0.01; ^3^*P* < 0.001. AF, aqueous fraction; CF, chloroform fraction; CQ, chloroquine; DW, distilled water; EF, ethylacetate fraction; US, *Urtica simensis.*

In congruent with crude extract, all solvent fractions ablated hemolysis in a dose-dependent manner (Figure 5). Eventhough there were some losses in PCV in the treatment groups (CE100 by 1.59 ± 4.72%, CF200 by 0.41 ± 2.56%, EF100 by 16.72 ± 4.66%, EF200 by 4.77 ± 4.14%, AF100 by 12.35 ± 2.47%, AF200 by 11.20 ± 1.71%,and AF400 by 5.23 ± 3.12%), these are incomparable (*P* > 0.05) to the PCV losses in the vehicle-treated group of mice (−20.07 ± 2.78%, −21.69 ± 8.82%, and −20.45 ± 5.53%), respectively. On the other side, group of animals treated with CF400 (1.2 6 ± 0.70%), EF400 (2.90 ± 3.38%), and standard treatment (3.72 ± 4.86%, 4.35 ± 2.96, and 4.05 ± 1.95, respectively) appreciate an increase in PCV. The standard drug, in comparison with all doses of the chloroform and ethyl acetate fraction, did not show notable differences in packed cell volume protection, but with AF indeed. Except negative controls, decrement and increment in PCV were insignificant compared to its baseline value.

#### 3.4.3. Effect of *U. simensis* Crude Extract and Solvent Fractions on Bodyweight and Temperature in the Suppressive Test

The effect of *U. simensis* on mouse bodyweight succeeding an infection was monitored, and the results conveyed that *P. berghei* infection inflicted a consequential bodyweight loss and basal body temperature in the infected but untreated group of mice compared to infected but treated animals (Table 4). Daily administration of *U. simensis* crude extract and chloroform fraction for four consecutive days significantly averted infected mice from bodyweight loss compared to negative control animals (Table 4), where bodyweight was contracted considerably postinfection. But neither AF nor EF was able to ablate bodyweight loss remarkably (*P* > 0.05) as compared to the negative control. Similarly, there was a significant variation in bodyweight between the baseline and at end of therapy. Both crude extract and CF's effect on bodyweight was in a dose-dependent approach; as the dose increases, its capacity to protect weight loss also increases in parallel. A group of mice was treated by US200, US400, CF400, and CQ25; in addition to preserve body weight at the hand, they can improve it by suppressing parasitemia efficiently.

As given in Table 4, the crude extract was able to significantly prevent the decrease in rectal temperature caused by *P. berghei* infection at a larger dose (US400, *P* < 0.05). But the lower and middle doses' capability to protect basal temperature loss fell short to reach a statistically significant level. Among all the three solvent fractions, only chloroform fraction was able to significantly avert the decrease in rectal temperature. All the three dose levels of the CF remarkably (*P* < 0.05) prevented the reduction in rectal temperature in relation to the control group. Furthermore, the reference drug also significantly (*P* < 0.01) prevented reduction in rectal body temperature in relation to the control group.

### 3.5. Antimalarial Curative Assay

**Figure 6:**
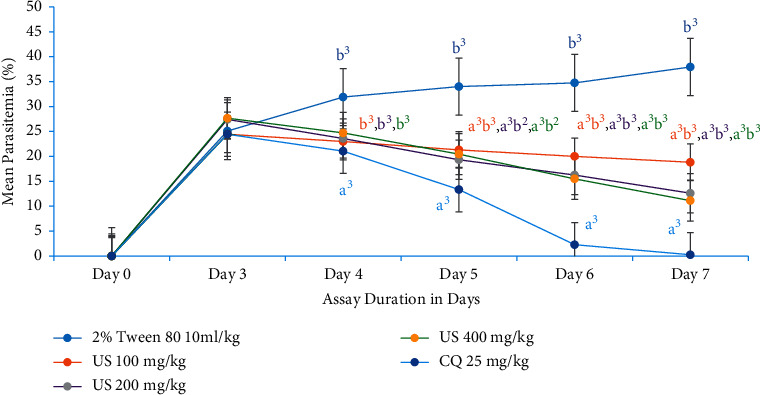
Evolution of parasitemia level of *P. berghei*-infected mice during treatment with hydromethanolic crude root extract of *U. simensis* in curative assay. Data are expressed as mean ± SEM (*n* = 6). ^a^Compared to negative control. ^b^Compared to the standard. ^1^*P* < 0.05; ^2^*P* < 0.01; ^3^*P* < 0.001. US, *Urtica simensis*; CQ, chloroquine.

Sanatory potential of the most active solvent fraction (chloroform fraction) and the hydromethanolic crude extract was evaluated in Rane's assay. The mean parasitemia in groups treated with hydromethanolic crude extract at US100 mg/kg ranged from 24.45 ± 1.07 to 18.82 ± 1.14, at US200 mg/kg, ranged from 27.42 ± 0.99 to 12.61 ± 0.74, and at US400 mg/kg, ranged from 27.67 ± 0.99 to 11.12 ± 0.60, while that of animals treated with chloroquine varied from 24.44 ± 0.62 to 0.26 ± 0.17. The mean parasitemia in the negative control group ranged from 25.08 ± 0.78 to 37.94 ± 1.30. All doses of crude extract demonstrated a significant mean percentage parasitemia difference when compared with the negative control group after the third dose (at day 5) (*P* < 0.001). But the activity of crude extract was significantly lower than the standard agent (Figure 6).

**Figure 7:**
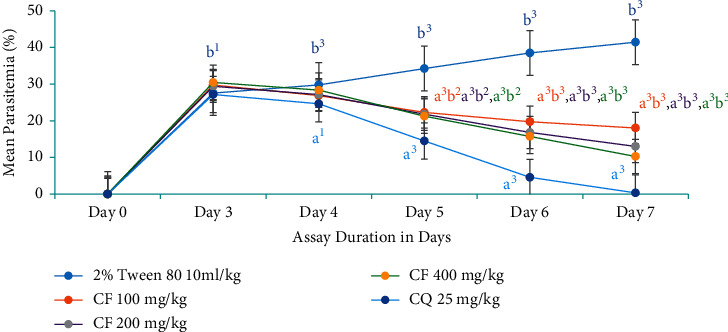
The effect of chloroform fraction of root of *U. simensis* on parasitemic level of *P. berghei*-infected mice in curative assay. Data are expressed as mean ± SEM (*n* = 6). ^a^Compared to negative control. ^b^Compared to the standard. ^1^*P* < 0.05; ^2^*P* < 0.01; ^3^*P* < 0.001. CF, chloroform fraction; CQ, chloroquine.

The mean parasitemia in groups treated with chloroform fraction at CF100 mg/kg ranged from 29.74 ± 0.82 to 18.02 ± 1.16, at CF200 from 29.40 ± 1.30 to 12.98 ± 0.66, and at CF400 from 30.45 ± 1.15 to 10.25 ± 0.8, while that of animals treated with chloroquine varied from 27.15 ± 1.01 to 0.31 ± 0.10. In congruent with the crude extract, all doses of chloroform fraction revealed a significant mean percentage parasitemia difference when compared with the untreated control group after the third dose (at day 5) (*P* < 0.001). But the activity of both crude extract and chloroform fraction was significantly lower (day 5, *P* < 0.01; day 6, *P* < 0.001; day 7, *P* < 0.001) than the standard agent (Figure 7).

**Figure 8:**
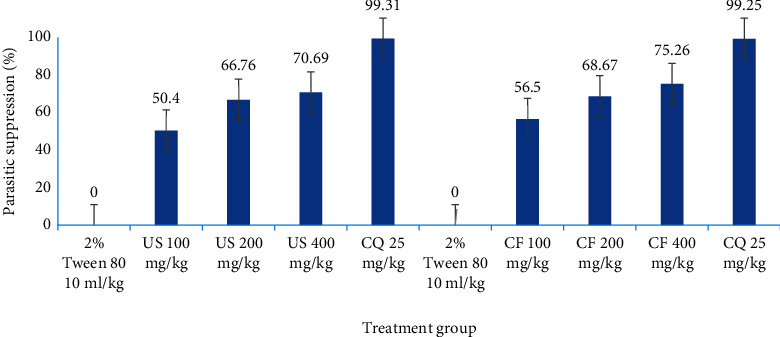
Parasitemic suppression capability of hydromethanolic crude root extract and chloroform fraction of *U. simensis* in *P. berghei*-infected mice in curative assay at day 7. Data are expressed as mean ± SEM (*n* = 6). US, crude extract of *Urtica simensis*; CF, chloroform fraction; CQ, chloroquine.

In vivo curative assay suppression outcomes for the hydromethanolic root extract of *U. simensis* using *Plasmodium berghei*-infected mice are shown in Figure 8. Crude extract of *U. simensis* roots decreased parasitemia level by 50.40%, 66.76%, and 70.69% for US100, US200 and, US400, respectively, compared to negative control mice. Similar to the crude extract, the result from chloroform fractions turned out that the parasitemia level was contracted by 56.5%, 68.67%, and 75.26% for CF100, CF200, and CF400, respectively, at day 7 compared to negative control mice which was reduced significantly (*P* < 0.001) as compared to vehicle-treated mice. Among all doses of crude extract and chloroform fractions, maximum inhibition (75.26%) of parasitemia levels was attained by 400 mg/kg dose of the chloroform fraction. The parasitemia inhibition seen with CQ25 was significantly (*P* < 0.001) higher than the crude extract and chloroform fraction. The positive control (chloroquine, 25 mg/kg) assayed in parallel with crude extract and CF reduced parasitemia by 99.31% and 99.25% with no observed mortality in the group after 30 days, respectively.

### 3.6. Effects of Crude Hydromethanolic Extract and Chloroform Fraction of *U. simensis* Roots on Mean Survival Time of Mice Infected with *P. berghei* in a Curative Assay

**Figure 9:**
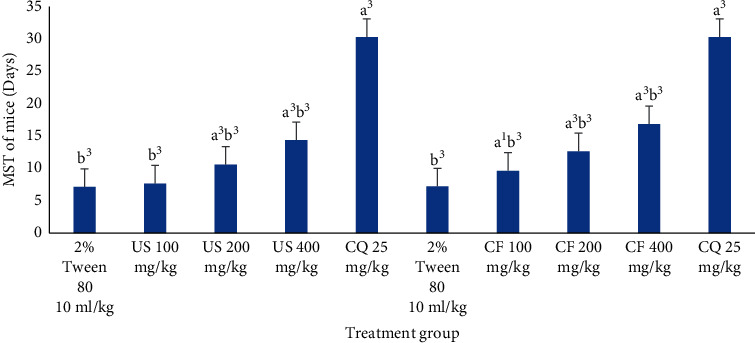
The effect of hydromethanolic crude root extract and chloroform fraction of *U. simensis* mean survival time in *P. berghei*-infected mice in a curative assay. Data are expressed as mean ± SEM (*n* = 6). ^a^Compared to negative control. ^b^Compared to the standard. ^1^*P* < 0.05; ^3^*P* < 0.001. CF, chloroform fraction; CQ, chloroquine; US, *Urtica simensis*.

Mean survival time analysis demonstrated that mice which received all doses of crude (except US100 (*P* < 0.05) and chloroform fraction (except CF100 (*P* < 0.05)) lived longer (with significant value *P* < 0.0001) than the mice in negative control (Figure 9). The mice treated with low (CF100), medium (CF200), and high (US400) doses of crude extract lived for 7.59 ± 0.21, 10.48 ± 0.42, and 14.22 ± 0.43 days, respectively). The survival rate of mice treated by lower (CF100), middle (CF200), and larger (CF400) doses of chloroform fractions was 9.55 ± 0.30, 12.53 ± 0.32, and 16.69 ± 1.07 days which was markedly prolonged (*P* < 0.001) than mice in the negative control group (Figure 9). Moreover, the group of mice treated with the standard agent was alive throughout the monitoring period (30.00 ± 0.00) days. Eventhough both crude extract and CF were able to prolong life of animals, but it is markedly lower (*P* < 0.001) than the standard agent ensued.

### 3.7. Effects of Crude Hydromethanolic Extract and Chloroform Fraction of *U. simensis* Roots on Bodyweight and Temperature of Mice Infected with *P. berghei* in a Curative Assay

Group of mice treated with the middle (US200 mg/kg per day, *P* < 0.05) and highest dose (US400 mg/kg per day, *P* < 0.001) of crude extract prevented reduction in bodyweight loss due to parasitemia. Whereas, the lowest dose (US100 mg/kg) fell flat in this regard (Table 5). Chloroform fraction at all doses (*P* < 0.05, *P* < 0.01, and *P* < 0.001) was able to ablate weight loss due plasmodial infection as compared to the negative control group of mice. Both crude extract and chloroform fraction at all doses were able to preclude basal body temperature reduction except at lower respective doses.

### 3.8. Effects of Crude Hydromethanolic Extract and Chloroform Fraction of *U. simensis* Roots on Packed Cell Volume of Mice Infected with *P. berghei* in a Curative Assay

**Figure 10:**
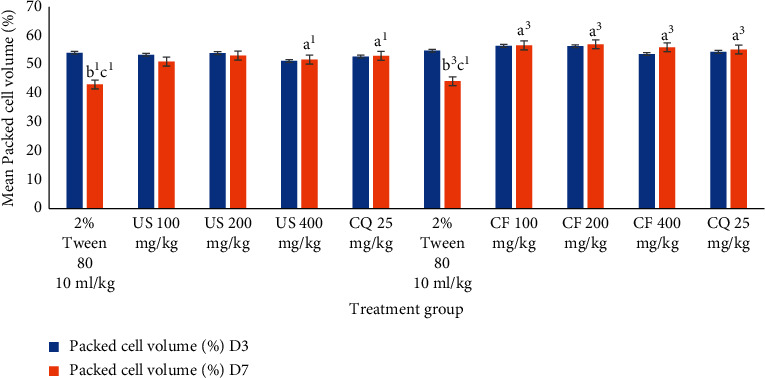
Effects of crude hydromethanolic extract and chloroform fraction of *U. simensis* roots on packed cell volume of mice infected with *P. berghei* in a curative assay. Data are expressed as mean ± SEM (*n* = 6). ^a^Compared to negative control. ^b^Compared to the standard. ^c^Compared to baseline. ^1^*P* < 0.05; ^3^*P* < 0.001. CF, chloroform fraction; CQ, chloroquine; D3, third day of *P. berghei* inoculation; D7, last day of the experiment; US, *Urtica simensis*.

Among hydromethanolic root crude extract doses, only the largest dose was able to prevent hemolysis as compared to the negative control group (*P* < 0.05). However, CF at all test doses was able to prevent hemolysis associated with high parasitemia (Figure 10) as compared to the respective negative control group appreciably (*P* < 0.001). But only negative controls showed a significant discrepancy from their baseline PCV (*P* < 0.05).

## 4. Discussion

Despite progress, malaria remains one of the world's most dangerous diseases. The malaria's global effect has forced a search for new antimalarial agents, fuelled by resistance to available and affordable antimalarial medications [46]. Antimalarial medications can be obtained from a variety of sources, with traditional medicinal herbs being one of the most reliable [47]. Artemisinin derivatives are a good illustration of how medicinal plants might help with antimalarial medication development [48]. Hitherto, *U. simensis* has not been studied for its antimalarial activity, eventhough used in Ethiopian folkloric medicine. As a result, the goal of this work is to evaluate the antimalarial potential of *U. simensis* extract and its solvent fractions as a new source for antimalarial treatment without jeopardizing its toxicological properties. Because herbal medicines are “natural,” they are frequently thought to be harmless; nonetheless, some products containing bioactive ingredients have the potential to induce side effects [49]. To that purpose, the current study focused on toxicity, which is a major problem with indigenous therapeutic preparations. From the acute toxicity assay of *U. simensis* root hydromethanolic extract in naive animals, the LD_50_ of *U. simensis* was found to be above 2000 mg/kg as no mortality or signs of toxic manifestation were observed, implying a wide safety margin and partly justifying the safety of the plant in traditional medicine as per OECD guideline no. 425 [32]. This safety profile was congruent with previous reports [26, 50]. Furthermore, it had no inherent ability to interfere with bodyweight, basal body temperature, or red blood cell evolution. In the present study, the antiplasmodial activity of the 80% hydroalcoholic extract of the root of *U. simensis* and its solvent fractions as well as their effect on the cardinal signs of *P. berghei-*infected mice such as bodyweight loss, hypothermia, and hemolytic anemia was investigated in rodents [51]. Standard in vivo antiplasmodial activity models such as Peter's test and Rane's assay models were employed. The typical preliminary paradigm for testing antiplasmodial effects in mice is Peter's test. Because of its capacity to establish a rodent model of malaria that is similar to human malaria infection, chloroquine-sensitive *P. berghei* ANKA was utilized in this work for inoculation and to predict antimalarial bioactivity [34, 35]. Despite the fact that chloroquine is no longer a first-line medicine for malaria therapy, it is employed in this study as a standard agent since the *Plasmodium* parasite used was chloroquine susceptible. Crude extract and CF at 100, 200, and 400 mg/kg inhibited parasitemia more effectively than ethylacetate and water fractions, and they were tested further in a curative assay.

In both early (14.27–79.24%) and established infections (50.40–75.26%), *U. simensis* reduced parasitemia, indicating possible suppressive and therapeutic benefits in malaria infection. To be deemed very good, good, or medium in terms of antimalarial efficacy in humans, an extract must inhibit parasitemia by at least 50% at doses of 100, 250, or 500 mg/kg/day [52]. This criterion places *U. simensis* in the top tier of antimalarial drug contenders.

Furthermore, the parasite inhibition displayed by 80% methanol extracts is equivalent to that observed in previous experiments using methanolic soybean seed extract (*Glycine max*) [53], ethanolic leaf and twig extract of *Faurea speciosa* [54], 80% methanolic root extract of *Echinops hoehnelii* [55], hydroethanolic stem bark extract of *Myrianthus libericus* [40], and aqueous fraction of *Schinus molle* seeds [56]. Nevertheless, the chemosuppression demonstrated by aqueous extract is less than that observed in a study using an aqueous extract of *Schinus molle* seeds [56]. The chloroform fraction inhibited parasitemia at a higher percentage than the crude extract and other solvent sections. This suggested that the possible potent active principles were soluble in the solvents accustomed for crude extract and solvent fractionation, and they are highly partitioned to a more semipolar to nonpolar solvent. Superficially, this might be due to the absence of alkaloids and flavonoids in AF and EF fractions. As a result, alkaloids, anthraquinones, phenol, tannins, and flavonoids found in this fraction may have attributed to the discovered antimalarial activity. Alkaloids [57], flavonoids [58], phenols [59], anthraquinones [60], and tannins [61] have all been implicated with antimalarial activity. This outcome is concordant with the efficacy of CF reported on CF of *Brucea antidysenterica* leaves [62], but higher with the low effect of CF reported on root of *Silene macrosolen* A. Rich (Caryophyllaceae) [63]. As evidenced by the chemosuppression observed during the early infection test, the extract shows blood schizonticidal action.

Additionally, the extract and fractions substantially increased the survival duration of animals in both protocols' dosage dependently, indicating that the overall reduction in parasitemia-related debilitating consequences in the treated groups may be attributed to the existence of metabolites. Anemia is one cardinal sign of malaria infection. The reduction in PCV that causes malarial anemia happens via an increase in the rate at which old red blood cells are destroyed and a decrease in hematopoiesis. *Plasmodium* enables to destroy both infected and uninfected red blood cells [64]. Mice treated with CF200 and CF400 totally redeemed the malaria-induced fall in PCV, in a comparable pattern to the reference medicines. The folklore applications of the herb in this regard are supported by this evidence, which lends validity to its antimalarial efficacy (Figures 5 and 10).

Rane's test is a typical antimalarial screening method for determining the effectiveness of extracts/drugs to cure existing infections. The parasitemia level in the infected control groups increased throughout the assay periods in this experiment. Regarding afflicted animals treated with the methanolic crude extract, chloroform fraction, and chloroquine phosphate, the mean percentage of parasitemia fell from the first day of therapy to its conclusion in an assay period in a dose-dependent way. This result unfolds that the involved extract is effective in schizonticidal activity even with limited doses to treat malarial infections.

In addition to having a strong suppressive effect on parasitemia, the crude extract and CF fraction increased the study mice's survival time at all test doses. Furthermore, CF (16.69 ± 1.07) has a longer survival duration than the crude extract (14.22 ± 0.43). This finding is consistent with earlier studies showing that animals with CF had a longer survival time [41].

Preliminary phytochemical screening may reveal some clues as to the type of the pharmacologically active substance in *U. simensis.*

There were alkaloids, triterpenoids, saponins, coumarins, and flavonoids identified in the *U. simensis* samples that were tested using established techniques. These kinds of chemicals have been shown to exhibit a curative effect against a variety of pathogens, which could support their historic use in the treatment of a variety of ailments, including malaria. Plant steroids, anthraquinones, and glycosides were found to be absent. This plant's phytochemical profile matches those of earlier studies [24, 26, 50]. The presence of secondary metabolites in medicinal plants has been associated to antiplasmodial effects in several investigations [12, 65–68].

Triterpenes' antiplasmodial effect is thought to be due to their modification of the cell membrane of nonparasitized erythrocytes, which prevents parasites from invading healthy RBCs [65, 66, 69]. *U. simensis* has also confirmed antioxidant activity which might explain its pharmacological activity and antimalarial activity [70].

Many antimalarial herbal medicines may have antiinfective effects not only by attacking the pathogen directly but also by activating the host's innate and adaptive defense mechanisms. The host's immune system is crucial in the total suppression or elimination of infections [71]. Furthermore, several plants have been shown to have antiplasmodial properties, either by increasing red blood cell oxidation or by limiting protein production [72]. Although the specific mechanism of action of this extract is unknown, it is possible that the plant functions through one or more of the routes indicated above, depending on the phytochemical content of the extract. Thus, the antiplasmodial activity discovered in this study could be attributed to a single or combination action of phytochemicals found in these crude extracts or fractionations, as shown by the results of this investigation.

## 5. Conclusion

In conclusion, the extract of *U. simensis* demonstrated antimalarial activities in vivo against murine malaria. Furthermore, this phytomedicine has been shown to have certain efficacies in terms of lowering cardinal symptoms including weight loss, temperature reduction, and hemolysis. As a result, traditional healers' use of this component of the plant for treating the aforementioned ailments has been substantiated.

### 5.1. Recommendation

- More research studies on the plant's antimalarial activity on diverse *Plasmodium* species and animal models are needed
- Bioassay guided isolation should be conducted to isolate the responsible active principle
- Elucidating the structure and mechanics of the active principle is recommended
- Detailed pharmacological and toxicological research studies are required to turn the active principles into lead compounds

## Figures and Tables

**Table 1:** Quantity and quality of hydromethanolic crude extract and solvent fractions.

Extracting solvent	Physical description of the extract	Yield (gm)	% yield (W/W)
80% hydromethanol	Dark blue powder	153	15.30
Chloroform	Brown powder	21.85	25.71
Ethyl acetate	Gummy dark brown semi powder	31.54	37.11
Aqueous	Reddish brown powder	31.01	35.31

**Table 2:** Qualitative phytochemical profiling of hydromethanolic crude extract.

Phytochemicals	Type of tests performed	Methanolic extract	Aqueous fraction	Ethylacetate fraction	Chloroform fraction
Alkaloids	Wagner's test	+	—	—	+
Phenolic compounds	Ferric chloride test	+	—	+	+
Glycosides	Keller–Killiani test	—	—	—	—
Plant steroids	Liebermann–Burchardt test	—	—	—	—
Anthraquinones	Borntrager's test	+	—	—	+
Terpenoids	Salkowski's test	+	+	+	−
Tannins	Braemer's test	+	+	+	+
Flavonoids	Alkaline reagent (NaOH) test	+	—	—	+
Saponins	Froth test	+	+	+	—

+, present; —, not detected.

**Table 3:** Parasitemia and survival time of *P. berghei*-infected mice treated with crude extract and solvent fractions of *U. simensis* in the 4-day suppressive test.

Extract	Animal group	% parasitemia level	% chemosuppression	Survival date
Crude extract	CON	41.03 ± 0.95	00.00 ± 00.00^b3^	7.50 ± 0.59^b3^
	US100	18.37 ± 0.39	55.23 ± 00.00^a3b3^	8.21 ± 0.44^b3^
	US200	13.13 ± 0.79	68.00 ± 00.00^a3b3^	11.00 ± 0.52^a3b3^
	US400	9.24 ± 0.49	77.48 ± 00.00^a3b3^	14.33 ± 0.49^a3b3^
	CQ25	00.00 ± 00.00	100.00 ± 00.00^a3^	30.00 ± 00.00^a3^

Chloroform fraction	CON	39.25 ± 2.34	00.00 ± 00.00^b3^	7.38 ± 0.42^b3^
	CF100	20.10 ± 0.94	48.79 ± 00^a3b3^	9.71 ± 0.31^a1b3^
	CF200	13.95 ± 0.44	64.46 ± 00.00^a3b3^	12.37 ± 0.41^a3b3^
	CF400	8.15 ± 0.51	79.24 ± 00.00^a3b3^	17.27 ± 0.98^a3b3^
	CQ25	00.00 ± 00.00	100.00 ± 00.00^a3^	30.00 ± 00.00^a3^

Ethylacetate fraction	CON	42.36 ± 0.94	00.00 ± 00.00^b3^	5.23 ± 0.18^b3^
	EF100	26.55 ± 0.96	37.32 ± 00.00^a3b3^	7.57 ± 0.25^a2b3^
	EF200	19.38 ± 0.53	54.25 ± 00.00^a3b3^	8.90 ± 0.27^a3b3^
	EF400	14.85 ± 0.83	64.94 ± 00.00^a3b3^	12.83 ± 0.67^a3b3^
	CQ25	00.00 ± 00.00	100 ± 00.00^a3^	30.00 ± 00.00^a3^

Aqueous fraction	DW	36.79 ± 2.01	00.00 ± 00.00^b3^	5.53 ± 0.10^b3^
	AF100	31.54 ± 0.55	14.27 ± 00.00^a3b3^	5.87 ± 0.06^b3^
	AF200	23.20 ± 0.31	50.08 ± 00.00^a3b3^	8.74 ± 0.43^a2b3^
	AF400	19.18 ± 0.60	58.73 ± 00.00^a3b3^	11.31 ± 1.02^a3b3^
	CQ25	00.00 ± 00.00	100 ± 00.00^a3^	29.83 ± 0.11^a3^

Data are expressed as mean ± SEM (*n* = 6). ^a^Compared to negative control. ^b^Compared to standard. ^1^*P* < 0.05; ^2^*P* < 0.01; ^3^*P* < 0.001. CON, negative control, received vehicle (2% Tween 80 10 ml/kg); DW, negative control, received distilled water 10 ml/kg; US, crude extract of *Urtica simensis*; CF, chloroform fraction; EF, ethylacetate fraction; AF, aqueous fraction; CQ, chloroquine. Numbers afterward letters in the second column refer to dose in mg/kg.

**Table 4:** Effect of *Urtica simensis* extract on bodyweight and rectal temperature of *P. berghei*-infected mice in Peter's test.

Treatment group	Temperature			Weight		
	D0	D4	% change in T	D0	D4	% change in Wt
CON	37.80 ± 0.15	33.86 ± 0.28	−10.40 ± 0.93^b3^	33.67 ± 0.49	28.55 ± 0.19	−15.21 ± 0.04^b3^
US100 mg/kg	37.89 ± 0.16	35.06 ± 0.40	−7.47 ± 1.19^b2^	33.19 ± 0.48	32.70 ± 0.55	−1.48 ± 1.40^a2^
US200 mg/kg	36.32 ± 0.38	33.92 ± 0.71	−6.58 ± 2.06^b2^	33.82 ± 0.31	34.40 ± 0.27	1.71 ± 0.88^a3^
US400 mg/kg	38.44 ± 0.24	37.22 ± 0.97	−3.19 ± 2.33^a1^	34.11 ± 0.21	35.67 ± 0.81	4.55 ± 2.02^a3^
CQ25 mg/kg	37.66 ± 0.14	38.26 ± 0.24	1.61 ± 0.75^a3^	34.23 ± 0.31	35.47 ± 0.53	3.65 ± 1.69^a3^
CON	37.7 ± 0.68	33.39 ± 1.28	−11.44 ± 4.55^b2^	34.31 ± 0.31	30.10 ± 1.08	−12.27 ± 3.83^b3^
CF100 mg/kg	37.23 ± 0.41	36.66 ± 0.79	−1.51 ± 1.68^a1^	33.56 ± 0.30	32.28 ± 0.66	−3.81 ± 1.67^a1^
CF200 mg/kg	37.24 ± 0.41	37.00 ± 0.54	−0.64 ± 0.98^a1^	33.57 ± 0.62	32.93 ± 0.88	−2.21 ± 2.72^a1^
CF400 mg/kg	37.08 ± 0.48	37.05 ± 0.53	−0.08 ± 1.29^a1^	33.11 ± 0.74	33.71 ± 0.36	1.92 ± 2.03^a3^
CQ25 mg/kg	37.61 ± 0.49	37.81 ± 0.36	0.53 ± 0.77^a2^	34.25 ± 0.31	35.29 ± 0.32	3.04 ± 0.77^a3^
CON	36.78 ± 0.24	32.90 ± 0.85	−10.54 ± 3.28^b1^	32.9 ± 0.76	30.78 ± 1.08	−6.44 ± 1.60^b2^
EF100 mg/kg	36.79 ± 0.40	32.06 ± 0.66	−12.79 ± 2.07^b2^	34.55 ± 0.40	35.59 ± 0.61	−3.01 ± 3.07
EF200 mg/kg	36.18 ± 0.47	32.13 ± 0.61	−11.19 ± 2.62^b1^	33.69 ± 0.55	29.8 ± 0.71	−11.55 ± 1.65^b3^
EF400 mg/kg	33.87 ± 0.99	32.21 ± 0.76	−4.90 ± 3.09	33.11 ± 0.67	30.34 ± 0.47	−8.34 ± 2.18^b1^
CQ25 mg/kg	37.08 ± 0.52	37.34 ± 0.43	0.70 ± 0.47^a1^	33.53 ± 0.80	34.03 ± 1.01	1.48 ± 1.34^a1^
DW10 ml/kg	36.78 ± 0.26	34.10 ± 0.93	−7.28 ± 3.08	32.87 ± 0.79	29.45 ± 1.50	−10.40 ± 6.60^b3^
AF100 mg/kg	36.74 ± 0.33	33.28 ± 0.83	−9.41 ± 1.94	33.96 ± 0.22	30.64 ± 0.53	−9.77 ± 1.74^b2^
AF200 mg/kg	36.14 ± 0.50	33.49 ± 0.98	−7.33 ± 2.91	33.64 ± 0.47	32.03 ± 0.40	−5.11 ± 2.05^b1^
AF400 mg/kg	35.60 ± 0.89	33.71 ± 0.82	−5.30 ± 2.12	33.53 ± 0.58	32.42 ± 0.63	−4.78 ± 3.04
CQ25 mg/kg	36.81 ± 0.33	38.67 ± 0.52	5.05 ± 1.20^a3^	34.17 ± 0.36	34.89 ± 0.54	2.03 ± 0.57^a1^

Data are expressed as mean ± SEM (*n* = 6). ^a^Compared to negative control. ^b^Compared to the standard. ^1^*P* < 0.05; ^2^*P* < 0.01; ^3^*P* < 0.001. CON, negative control, received vehicle (2% Tween 80 10 ml/kg); DW, negative control, received distilled water 10 ml/kg; US, crude extract of *Urtica simensis*; CF, chloroform fraction; EF, ethylacetate fraction; AF, aqueous fraction; CQ, chloroquine.

**Table 5:** Effects of crude hydromethanolic extract and chloroform fraction of *U. simensis* roots on bodyweight and temperature of mice infected with *P. berghei* in a curative assay.

Treatment group	Temperature			Weight		
	D3	D7	% change in T	D0	D4	% change in Wt
CON	37.57 ± 0.31	33.80 ± 0.36	−10.02 ± 0.82^b3^	33.67 ± 0.50	30.04 ± 0.54	−10.78 ± 2.11^b2^
US100 mg/kg	37.02 ± 0.57	35.03 ± 1.03	−5.45 ± 1.75^b1^	31.14 ± 1.21	29.44 ± 1.26	−5.45 ± 2.98^b2^
US200 mg/kg	37.44 ± 0.48	36.18 ± 0.95	−3.41 ± 1.80^a1^	29.23 ± 1.21	28.41 ± 0.88	−2.80 ± 2.46^a1^
US400 mg/kg	37.50 ± 0.79	37.82 ± 0.42	0.75 ± 1.53^a2^	29.63 ± 0.92	31.25 ± 1.07	5.46 ± 2.87^a3^
CQ25 mg/kg	37.03 ± 0.47	37.56 ± 0.09	1.49 ± 1.25^a3^	33.61 ± 0.56	34.49 ± 0.38	2.61 ± 2.42^a2^
CON	37.07 ± 0.68	35.57 ± 0.78	−9.46 ± 0.80^b3^	29.83 ± 1.25	27.61 ± 0.97	−7.44 ± 1.36^b3^
CF100 mg/kg	36.56 ± 0.73	34.67 ± 0.82	−5.16 ± 1.20^b2^	29.82 ± 1.09	28.20 ± 1.24	−5.43 ± 0.93^b2^
CF200 mg/kg	36.55 ± 0.69	35.29 ± 0.47	−3.32 ± 1.85^a1b1^	29.74 ± 1.24	29.72 ± 1.20	−0.06 ± 1.09^a1^
CF400 mg/kg	37.20 ± 0.42	36.64 ± 0.74	−1.53 ± 1.21^a2^	29.33 ± 1.03	29.89 ± 1.09	1.90 ± 2.18^a2^
CQ25 mg/kg	37.60 ± 0.36	38.40 ± 0.49	2.14 ± 1.27^a3^	30.78 ± 1.11	31.62 ± 1.01	2.73 ± 1.15^a3^

Data are expressed as mean ± SEM (*n* = 6). ^a^Compared to negative control. ^b^Compared to the standard. ^1^*P* < 0.05; ^2^*P* < 0.01; ^3^*P* < 0.001. CON, negative control, received vehicle (2% Tween 80 10 ml/kg); US, crude extract of *Urtica simensis*; CF, chloroform fraction; CQ, chloroquine.

## Data Availability

The data used to support the findings of this study are available from the corresponding author upon request.

## Abbreviations

ANOVA: Analysis of variance
EPHI: Ethiopian Public Health Institute
ICLAS: International Council for Laboratory Animal Science
LD_50_: Median lethal dose
OECD: Organization for Economic Cooperation and Development
PCV: Packed cell volume
SPSS: Statistical Package for Social Sciences.
